# Estrogen-decreased hsa_circ_0001649 promotes stromal cell invasion in endometriosis

**DOI:** 10.1530/REP-19-0540

**Published:** 2020-07-06

**Authors:** Qi Li, Na Li, Hengwei Liu, Yu Du, Haitang He, Ling Zhang, Yi Liu

**Affiliations:** 1 Department of Obstetrics and Gynecology, Union Hospital, Tongji Medical College, Huazhong University of Science and Technology, Wuhan, China; 2 Department of Obstetrics and Gynecology, Zhongnan Hospital of Wuhan University, Wuhan University, Wuhan, China; 3 Department of Obstetrics and Gynecology, Tongji Hospital, Tongji Medical College, Huazhong University of Science and Technology, Wuhan, China

## Abstract

Endometriosis (EMs) is an estrogen (E_2_)-dependent inflammatory disorder. Although EMs is considered a benign disease, it presents with malignant characteristics, such as migration and invasion. An increasing number of studies have shown that aberrantly expressed circular RNAs (circRNAs) play an essential role in disease development and progression. However, the mechanisms by which circRNAs exert their pathological effects in EMs remain unclear. Hsa_circ_0001649, a novel cancer-associated circRNA, has been previously reported to be downregulated in several cancer types and related to cell migration and invasion. In the present study, real-time PCR (qRT-PCR) was carried out to measure hsa_circ_0001649 levels in human tissues, human primary endometrial stromal cells (ESCs) and a human endometrial stromal cell line (ThESCs). Matrix metalloproteinase 9 (MMP9) levels in ESCs and ThESCs were assessed by qRT-PCR and Western blotting, and the migration and invasion capacities of ThESCs were evaluated by transwell assay. As a result, hsa_circ_0001649 expression was significantly decreased in ectopic and eutopic endometrial samples compared with that in normal endometrial samples. E_2_ decreased hsa_circ_0001649 expression but increased MMP9 expression in ESCs and ThESCs. Furthermore, ThESCs were more invasive under E_2_ stimulation. However, these effects disappeared when ICI or hsa_circ_0001649 transfection was used. Collectively, our findings reveal that decreased hsa_circ_0001649 expression plays a role in E_2_-increased MMP9 expression through E_2_ receptors (ERs), which have critical functions in EMs.

## Introduction

Endometriosis (EMs), which is histologically defined by the presence of endometrial tissue outside the endometrial cavity, is an estrogen (E_2_)-dependent inflammatory disorder associated with variable phenotypic and symptomatic presentations (Yilmaz & Bulun 2019). EMs is estimated to occur in at least 10% of reproductive-aged women, 40% of infertile women and 90% of women with pelvic pain (Kodaman 2015). Despite extensive research and various theories, the pathogenesis of EMs remains controversial (Harada* et al.* 2001). Moreover, EMs is an enigma due to delayed diagnosis and ineffective treatment (Kvaskoff* et al.* 2015). Therefore, additional basic research is needed to enhance our understanding of the etiology of EMs and identify new diagnosis and treatment options.

An increasing number of studies have shown that noncoding RNAs (ncRNAs) are functionally important for normal development and physiology as well as pathology (Esteller 2011). NcRNAs, including miRNA, long ncRNAs (lncRNAs) and circular RNAs (circRNAs) (Anastasiadou* et al.* 2018), are functional RNAs transcribed from DNA but are mostly not translated into proteins (Cech & Steitz 2014). Recently, several studies have focused on the roles of miRNAs and lncRNAs in Ems (Ferlita* et al.* 2018, Panir* et al.* 2018). We also previously found that the lncRNA MALAT1 mediates hypoxia-induced pro-survival autophagy in endometrial stromal cells (ESCs) in Ems (Liu* et al.* 2019) and that MALAT1 acts as a miRNA sponge for miR200, which is involved in the E_2_-induced epithelial-mesenchymal transition (EMT) in Ems (Du* et al.* 2019). However, few studies have focused on the newly discovered circRNAs in EMs. In contrast to linear RNAs, circRNAs are characterized by covalently linked terminals, high stability in the circulation, and abundant expression, rendering them ideal biomarkers for diagnostic, prognostic, and therapeutic response predictions (Qu* et al*. 2015). Moreover, circRNAs play a key regulatory role in gene expression at the transcriptional and posttranscriptional levels (Greene* et al.* 2017). Although the mechanism of action of circRNAs is unclear, several circRNAs have been reported to act as ‘miRNAs sponges‘ to regulate miRNA activity (Hansen* et al.* 2013), to interact with RNA-binding proteins (RBPs) (Zang* et al.* 2020), or to bind to RNA-pol II to regulate transcription (Li* et al.* 2015) and even translation into specific proteins in some particular cases (Pamudurti* et al.* 2017). Regardless, research to date on the mechanism of circRNA in EMs is still in its infancy. Of the few studies that have investigated EMs and circRNAs, most have focused only on differences in circRNA expression in EMs and have not examined how differentially expressed circRNAs function in EMs (Shen* et al.* 2018, Xu* et al.* 2018*a*,*b*, Zhang* et al.* 2018, 2019, Wang* et al.* 2019). Thus, more effort is required to determine the mechanisms by which circRNAs exert their pathological effects on EMs.

Although EMs is considered a benign disease, it has biological behaviors similar to malignant tumors, such as invasion, distant metastasis, and recurrence. Hsa_circ_0001649, a transcriptional product of the tumor suppressor gene SNF2 histone linker PHDRINGhelicase (SHPRH) (Wang* et al.* 2018), is a novel cancer-associated circRNA that exhibits decreased expression in several cancer types, including cholangiocarcinoma, hepatocellular carcinoma and colorectal cancer (Xing* et al.* 2018). Qin* et al.* (2016) confirmed that hsa_circ_0001649 knockdown in vitro increases expression of matrix metalloproteinases (MMPs, including MMP9, MMP10 and MMP13) in human hepatoma cells and promotes tumor invasion and metastasis. MMP9 is an important member of the MMPs family involved in cell invasion and metastasis (van Kempen & Coussens 2002). Moreover, MMP9 is highly expressed in endometriosis lesions (Pino* et al.* 2009), and in our previous research, we found that MMP9 promotion through the Wnt/β-catenin pathway under E_2_ regulation may contribute to the pathophysiology of Ems (Zhang* et al.* 2016). However, whether hsa_circ_0001649 is involved in E_2_-mediated MMP9 upregulation in EMs remains unknown.

In the present study, we aimed to determine whether hsa_circ_0001649 expression levels differ among eutopic, ectopic, and normal endometrial samples and whether hsa_circ_0001649 is involved in E_2_-mediated MMP9 upregulation in EMs. Our study provides novel insight into the circRNA-related pathogenesis of EMs.

## Materials and methods

### Tissue collection

Paired eutopic (*n* = 40) and ectopic (*n* = 40) endometrial samples were collected from patients with ovarian EMs undergoing laparoscopy. These patients were 21–37 years of age and classified as having endometriosis at stages I–IV according to the American Society for Reproductive Medicine (ASRM) criteria (American Society for Reproductive Medicine 1997). Normal endometrial samples (*n* = 101) were collected by curettage from tubal infertility patients 21–39 years of age without EMs. RNA was extracted from a portion of the normal endometrial samples (*n* = 40) to detect the expression levels of hsa_circ_0001649, and ESCs were extracted from the remaining normal endometrial samples (*n* = 61) for subsequent experiments. Patients with pelvic inflammatory disease, adenomyosis and dysfunctional uterine bleeding were excluded. The participants had not received hormonal therapy for ≥6 months before the surgical procedure. Informed consent was obtained with approval from the Local Ethics Committee of Tongji Medical College, Huazhong University of Science and Technology. All patients signed an informed consent form before entering the study. All samples were obtained during the proliferative phase of the regular menstrual cycle to avoid the effect of progesterone according to their last menstrual period and further confirmed histologically by Noyes criteria (Noyes & Haman 1953, Noyes* et al.* 1975).

### Cell culture

Human primary ESCs were isolated from the normal endometrium of patients with tubal infertility without EMs. The tissues were minced with scissors and digested enzymatically with collagenase type II (0.1%; Sigma-Aldrich) for 40 min at 37°C with constant agitation. Then, the tissue pieces were sequentially filtered through sterile 400-µm and 100-µm sieve wires to remove undigested tissue and epithelial cells, and the filtrate was centrifuged at 129 ***g*** for 5 min to separate the ESCs from the collagenase. The ESC pellets were suspended in Red Blood Cell Lysis Buffer (C3702, Beyotime, Jiangsu, China) for 5 min to remove erythrocytes. The cell suspensions were centrifuged at 129 ***g*** for 5 min, and the pelleted ESCs were resuspended in Dulbecco’s Modified Eagle’s Medium (DMEM)/F-12 medium containing 10% fetal bovine serum (FBS). The ESCs were placed in a culture flask and incubated in 5% CO_2_ at 37°C.

A human endometrial stromal cell line (ThESCs) was purchased from American Type Culture Collection (CRL-4003; ATCC) and cultured in the same medium and environment as the ESCs.

### Hormone treatment

E_2_ (E-2758, Sigma–Aldrich) and an E_2_ receptor (ER) antagonist ICI 182,780 (CAS 129453-61-8, Cayman Chemicals) were dissolved in DMSO. After incubation in serum-free and phenol red-free DMEM/F-12 for 24 h, ESCs and ThESCs were treated with different concentrations (0, 10^−11^, 10^−10^, 10^−9^, 10^−8^, and 10^−7^ mol/L) of E_2_ and incubated for different durations (0, 12, 24, and 48 h). The medium was changed to fresh complete medium every 24 h.

### Quantitative RT-PCR (qRT-PCR)

Total RNA was extracted from the tissue samples and cultured cells using TRIzol reagent (VazymeBiotech, Nanjing, China). cDNA was synthesized with HiScript II Q RT SuperMix (VazymeBiotech), qRT-PCR was performed using a StepOnePlus real-time PCR system (Applied Biosystems) with the preset PCR program, and *GAPDH* was used as an internal control to quantify mRNA expression.

The qRT-PCR primer sequences are shown in Supplementary Table 1 (see section on supplementary materials given at the end of this article), where the different primers used to quantify hsa_circ_0001649 are cited from a study by Wang* et al.* (2018). The primers for hsa_circ_0001649 are located in the flanking region of the back-splice site where exon 26 and exon 29 are linked, as shown in Supplementary Fig. 1 (Zhong* et al.* 2018). The qRT-PCR products of hsa_circ_0001649 were analyzed by Sanger sequencing to confirm the back-splice junction of hsa_circ_0001649. The PCR cycling conditions were 95°C for 30 s, followed by 95°C for 10 s, 60°C for 30 s, and a dissociation program of 95°C for 15 s, 60°C for 30 s and 95°C for 15 s. The relative expression levels of the target genes were calculated by the 2^−ΔΔCt^ method.

### Western blotting

The total proteins extracted from the cultured ESCs and ThESCs were quantified with a BCA protein assay kit (P0010S; Beyotime). The protein samples were incubated for 10 min at 95°C; then, proteins (30 mg) were subjected to 10% SDS-PAGE and transferred to PVDF membranes (0.45-µm pore size; Millipore). The blots were incubated with 5% skim milk in Tris-buffered saline containing 0.05% Tween 20 at room temperature for 1 h and then incubated overnight at 4°C with rabbit polyclonal anti-MMP9 (1:1000; 10375-2-AP; Proteintech, Rosemont, Illinois, USA) or rabbit monoclonal anti-GAPDH (1:1000; ab181602; Abcam) primary antibodies. Subsequently, the membranes were washed with TBST and incubated with a secondary anti-rabbit antibody (1:4000; Affinity, USA) for 1 h at room temperature. The proteins were visualized by the enhanced chemiluminescence method (WBKLS0500; Millipore) according to the manufacturer’s recommendations.

### Hsa_circ_0001649-knockdown plasmid construction

ShRNA against hsa_circ_0001649 (GenePharma, Shanghai, China) was designed to target the hsa_circ_0001649 BSJ region and cloned into a pGPU6-GFP-Neo vector for use. The shRNA sequences were as follows: 5′-CACCGTGGCTGCCCTTCTCTCAGCTTCAAGAGAGCTGAGAGAAGGGCAGCCATTTTTTG-3′ (sense) and 5′-GATCCAAAAAATGGCTGCCCTTCTCTCAGCTCTCTTGAAGCTGAGAGAAGGGCAGCCAC-3′ (antisense).

ShRNA for hsa_circ_0001649 was synthesized in the nucleus after being transfected into cells and subsequently transported to the cytoplasm. With the Dicer-containing complex, the stem-loop structure in the shRNA was removed and siRNA was formed. The antisense strand of the siRNA was then incorporated into the RNA-induced silencing complex (RISC) after chain unwinding. Hsa_circ_0001649 was cleaved by the RISC, with the siRNA binding to the BSJ region in a base complementary manner, and subsequently degraded (as shown in Supplementary Fig. 2A). Control shRNA was constructed with the same vector as the hsa_circ_0001649 shRNA construct and sequences that are not complementary to the BSJ region. In addition, these sequences have been proven to have no effect on normal human cells (Cheng* et al.* 2010, Li* et al.* 2013, Wang* et al.* 2015). The shNC sequences were as follows: 5′-CACCGTTCTCCGAACGTGTCACGTCAAGAGATTACGTGACACGTTCGGAGAA TTTTTTG-3′ (sense) and 5′-GATCCAAAAAATTCTCCGAACGTGTCACGT AATCTCTTGACGTGACACGTTCGGAGAAC-3′ (antisense) (as shown in Supplementary Fig. 2B).

### Hsa_circ_0001649-overexpression plasmid construction

A pcDNA3.1 vector that can effectively overexpress circRNA was used for hsa_circ_0001649 overexpression (Liang & Wilusz 2014), and an empty plasmid vector was used as a control (GenePharma). CircRNA overexpression vectors include two reverse complementary sequences (an upstream fragment and a downstream fragment, as shown in Supplementary Fig. 3) and the circRNA sequence. The upstream and downstream reverse complementary sequences produce a stable RNA hairpin structure after transcription to promote circRNA circularization. The full-length hsa_circ_0001649 sequence was inserted between the reverse complementary sequences with restriction enzyme cutting sites (NheI, HindIII) and linked to the vector to construct an hsa_circ_0001649-overexpression vector (Supplementary Fig. 3). The overexpression efficiency of hsa_circ_0001649 was examined by qRT-PCR.

### Cell transfection

ThESCs were seeded in six-well plates, grown to 60–80% confluence, and transfected with the indicated plasmids using Neofect (Neofect Biotech, Beijing, China). The cells were transfected for 48 h and then collected for subsequent analyses.

### Transwell assays

Transwell units (24-well plates, insert membranes with 8-µm pores; Corning Costar) were coated with or without Matrigel to investigate the cell migration and invasion capacities, respectively. In each well, transfected ThESCs were resuspended in 200 μL of serum-free DMEM/F12 medium with or without 17β-E_2_ and placed in the upper chamber, and 500 μL of complete medium was added to the lower chamber. The transwell units were incubated in 5% CO_2_ at 37°C for 24 h, and the cells and Matrigel were removed from the upper membrane surface and stained with 0.1% crystal violet for 20 min at 37°C. The number of cells on the underside of the membrane was counted under a light microscope (Olympus). Five randomly selected fields were counted per insert.

### Statistical analysis

All data are shown as the mean ± s.d. Statistical analyses were performed using GraphPad Prism 5.01 software (GraphPad Software) and SPSS 19.0 statistical software (SPSS). The Kruskal–Wallis test was used to compare non-normally distributed variables. For normally distributed variables, an unpaired Student’s *t*-test was used to determine significant differences between two groups, and one-way ANOVA was used for comparisons between three or more groups. *P* < 0.05 was considered statistically significant. All data were obtained from ≥3 independent experiments.

## Results

### Characterization and expression of hsa_circ_0001649 in EMs

**Figure 1:**
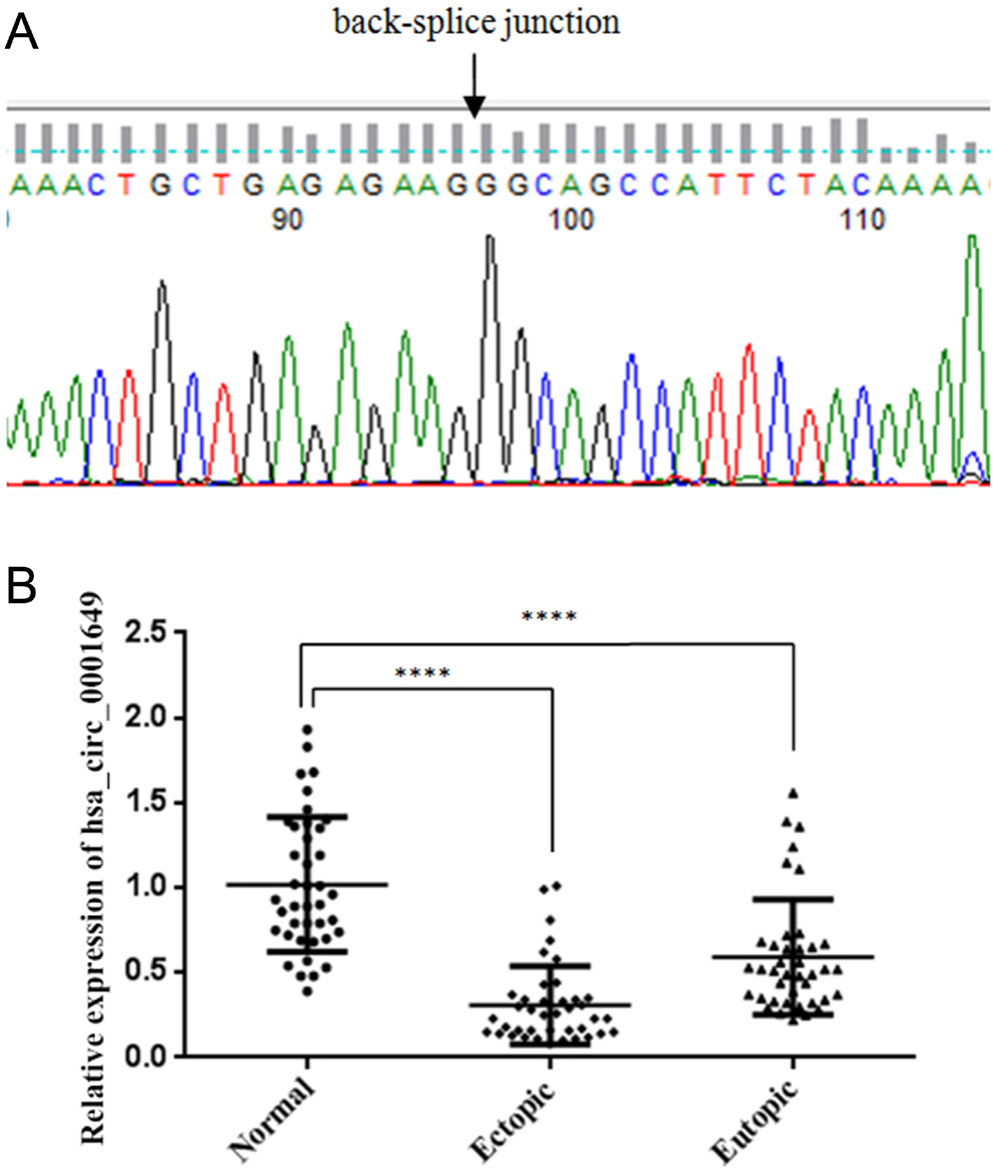
Characterization and expression of hsa_circ_0001649 in EMs. (A) The Sanger sequence of hsa_circ_0001649 qRT-PCR products. (B) The relative expression levels of hsa_circ_0001649 in eutopic (*n* = 40) and ectopic (*n* = 40) endometrial specimens from patients with ovarian endometriosis (EMs) and normal endometrial (*n* = 40) specimens were measured by qRT-PCR. The data are expressed as the mean ± s.d. ****P < 0.0001 by the Kruskal–Wallis test.

To determine whether hsa_circ_0001649 is involved in the pathogenesis of EMs, we acquired the hsa_circ_0001649 products amplified by qRT-PCR, which were used for Sanger sequencing, and the result was consistent with the sequence in CircBase (Fig. 1A), revealing the expression of hsa_circ_0001649 in EMs tissue. Then, we measured the relative expression of hsa_circ_0001649 in 40 paired eutopic and ectopic endometrial samples from patients with EMs and 40 normal endometrial samples by qRT-PCR. As indicated in Fig. 1B, compared with that in the normal endometrium, the expression level of hsa_circ_0001649 was significantly decreased in ectopic (*P* < 0.0001) and eutopic (*P* < 0.0001) endometrium samples from EMs patients.

### E**_2_** downregulates hsa_circ_0001649 through estrogen receptors (ERs) in ESCs and ThESCs

**Figure 2:**
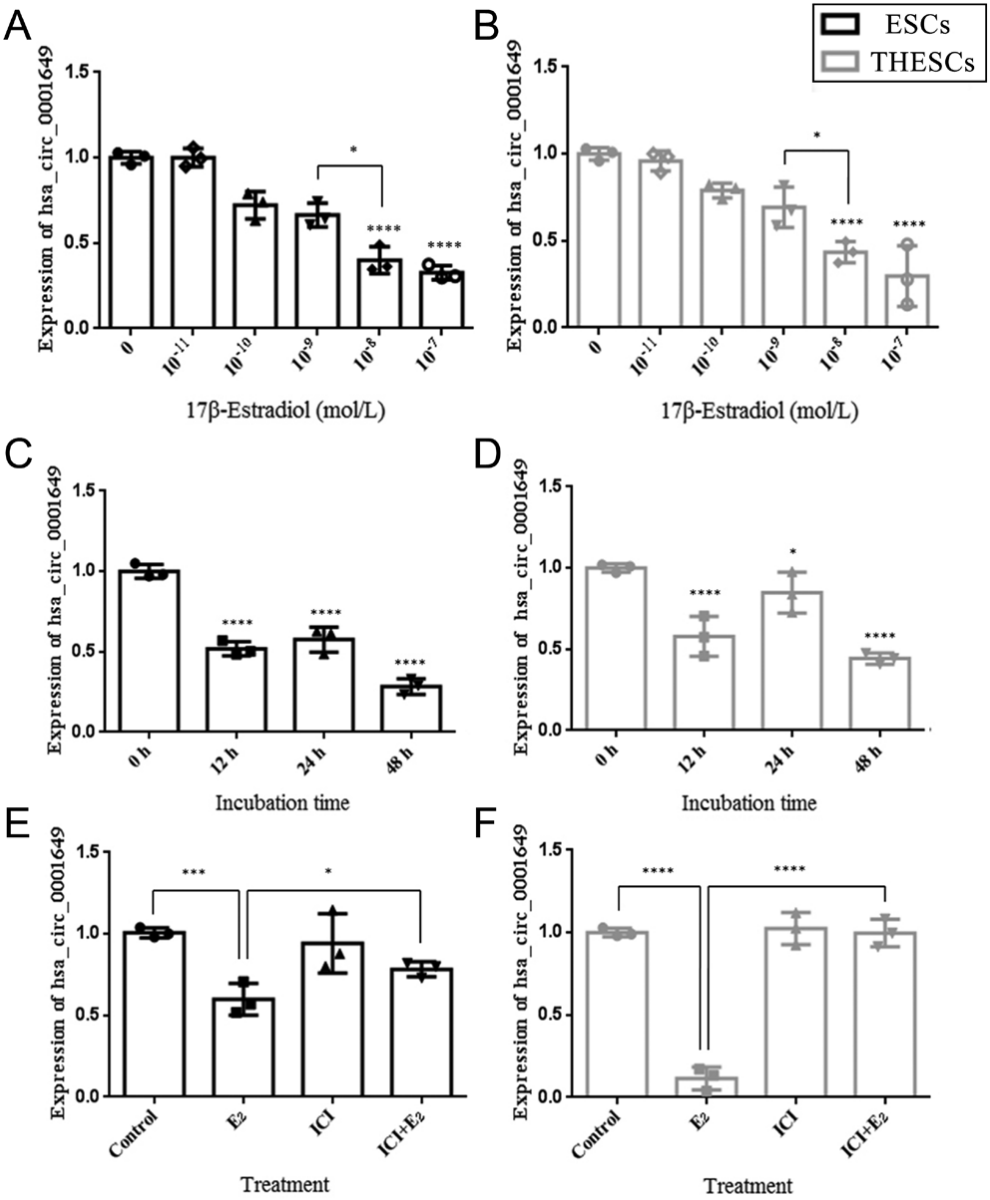
Hsa_circ_0001649 expression is regulated by estrogen (E2) in human primary endometrial stromal cells (ESCs) and a human endometrial stromal cell line (ThESCs). (A and B) Hsa_circ_0001649 expression in ESCs and ThESCs was determined by qRT-PCR following treatment with E_2_ at a series of concentrations (0, 10^−11^, 10^−10^, 10^−9^, 10^−8^, and 10^−7^ mol/L). (C and D) Hsa_circ_0001649 expression in ESCs and ThESCs was determined following 10^−8^ mol/L E_2_ treatment for the indicated durations (0, 12, 24, and 48 h). (E and F) Hsa_circ_0001649 expression was determined following vehicle, 10^−8^ mol/L E_2_, 10^−6^ mol/L estrogen receptor antagonist ICI 182,780 (ICI) and 10^−8^ mol/L E_2_ + 10^−6^ mol/L ICI stimulation for 48 h. The data are expressed as the mean ± s.d. of three independent experiments. *P < 0.05; ***P < 0.001 and ****P < 0.0001 by one-way ANOVA.

As EMs is an E_2_-dependent disease, we investigated whether the lower hsa_circ_0001649 expression in EMs patients than that in the normal endometrium is caused by E_2_. Both ESCs and ThESCs were treated with various gradient concentrations of 17β-E_2_ (0, 10^−11^, 10^−10^, 10^−9^, 10^−8^, and 10^−7^ mol/L) for 48 h. We observed that E_2_ decreased hsa_circ_0001649 expression in ESCs and ThESCs in a concentration-dependent manner, with significant inhibition at 10^−8^ mol/L and 10^−7^ mol/L (*P* < 0.0001) (Fig. 2A and B). The physiological concentration of E_2_ in women ranges from approximately 10^−10^ mol/L to 10^−9^ mol/L, whereas the endometriotic intra tissue E_2_ concentration is approximately 10^−8^ mol/L (Huhtinen* et al.* 2012). We compared hsa_circ_0001649 expression in cells treated with 10^−9^ mol/L E_2_ with that in cells treated with 10^−8^ mol/L E_2_ and found significant decreases in cells under 10^−8^ mol/L E_2_ stimulation (*P* < 0.05) (Fig. 2A and B). Therefore, together with the results obtained from the concentration gradient experiments, we selected 10^−8^ mol/L E_2_ stimulation for our subsequent experiments. ESCs and ThESCs were cultured with 10^−8^ mol/L E_2_ for different durations (0, 12, 24, or 48 h). As shown in Fig. 2C, compared with that in unstimulated control cells, a time-dependent decrease in hsa_circ_0001649 expression was observed, and the lowest level was observed at 48 h (*P* < 0.0001). Similar results were obtained with the ThESCs, with a significant reduction in the expression of hsa_circ_0001649 being observed at 48 h (*P* < 0.0001) (Fig. 2D).

To further determine whether the E_2_-mediated regulation of hsa_circ_0001649 expression was dependent on ERs, ESCs and ThESCs were divided into the following four experimental groups: group 1, control group (no treatment); group 2, treated with 10^−8^ mol/L E_2_; group 3, treated with 10^−6^ mol/L ICI; and group 4, treated with 10^−6^ mol/L ICI in conjunction with 10^−8^ mol/L E_2_. As shown in Fig. 2E and F, the E_2_-mediated inhibition of hsa_circ_0001649 expression was antagonized by ICI in the ESCs (*P* < 0.05) and ThESCs (*P* < 0.0001). These results suggest that E_2_ inhibits the expression of hsa_circ_0001649 in ESCs and ThESCs via ERs.

### E**_2_** increases MMP9 expression in ESCs and ThESCs through hsa_circ_0001649

**Figure 3:**
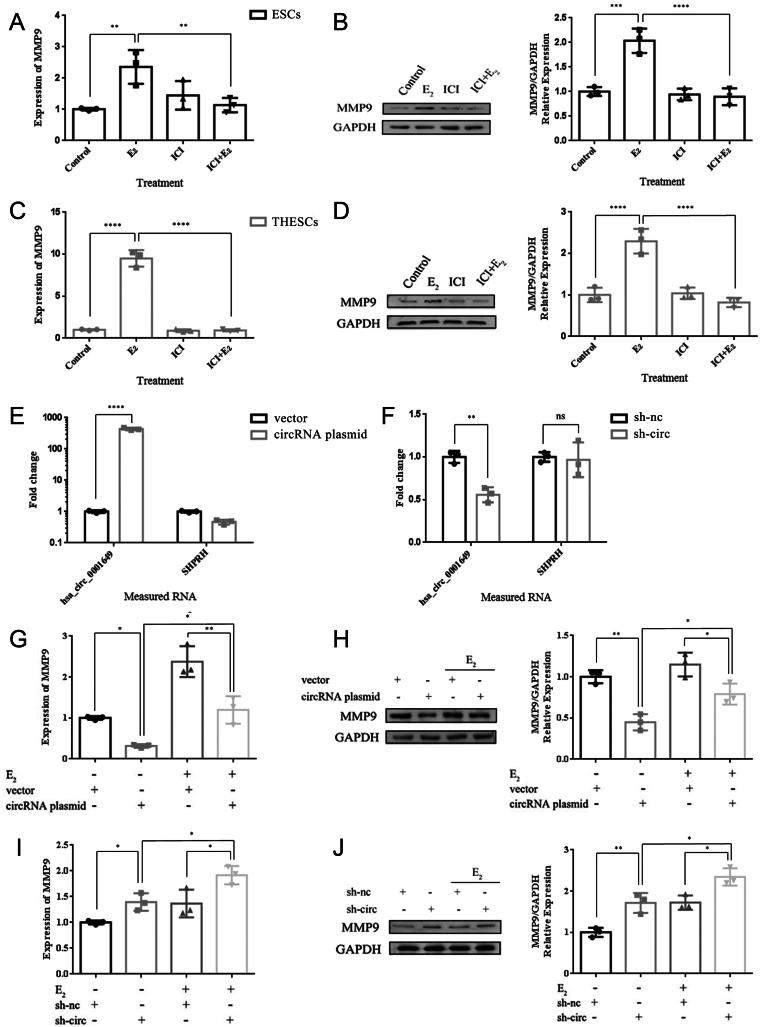
Effects of hsa_circ_0001649 on estrogen (E_2_)-mediated MMP9 expression in ESCs and ThESCs. (A, B, C and D) MMP9 mRNA and protein expression in ESCs and ThESCs treated with vehicle-only controls, E_2_, ICI and E_2_ + ICI. (E) hsa_circ_0001649 and SHPRH (negative control) expression in ThESCs transfected with a pcDNA3.1 vector overexpressing hsa_circ_0001649 (circRNA plasmid) or an empty vector (vector). (F) hsa_circ_0001649 and SHPRH expression in ThESCs transfected with hsa_circ_0001649 shRNA (sh-circ) or control shRNA (sh-nc). (G and H) MMP9 mRNA and protein expression in ThESCs transfected with vector, circRNA plasmid, vector + E_2_, and circRNA plasmid + E_2_. (I and J) MMP9 mRNA and protein expression in ThESCs transfected with sh-nc, sh-circ, sh-nc + E_2_, and sh-circ + E_2_. RNA expression was measured by qRT-PCR and protein expression by Western blotting; the results are represented as the fold change compared to the vehicle-only controls. The data are presented as the mean ± s.d. of three independent experiments. *P < 0.05; **P < 0.01; ***P < 0.0001; and ****P < 0.0001 by one-way ANOVA and Student’s *t*-test. n.s., not significant.

To investigate the action of E_2_ on MMP9, ESCs and ThESCs were treated with E_2_, ICI, and ICI in conjunction with E_2_ and compared with vehicle-only controls. Western blot and qRT-PCR analyses demonstrated that E_2_-mediated expression of MMP9 was antagonized by ICI at the protein and mRNA levels in both ESCs (*P* < 0.01) (Fig. 3A and B) and ThESCs (*P* < 0.0001) (Fig. 3C and D). Altogether, these results indicate that E_2_ increases MMP9 expression via ERs.

Then, we further investigated whether hsa_circ_0001649 is involved in E_2_-mediated MMP9 expression. Hsa_circ_0001649 expression in ThESCs was effectively knocked down by the shRNA specifically targeting the splice junction of hsa_circ_0001649 (sh-circ) or increased by an hsa_circ_0001649-forming plasmid (circRNA plasmid) (Fig. 3E and F). qRT-PCR and Western blot analyses showed that knocking down the expression of hsa_circ_0001649 under the E_2_ conditions resulted in the maximum MMP9 expression, while the overexpression of hsa_circ_0001649 not only inhibited MMP9 expression but also reversed the promotional effect of E_2_ on MMP9 (Fig. 3G, H, I and J). These results suggest that hsa_circ_0001649 is involved in E_2_-mediated MMP9 expression.

### Hsa_circ_0001649 influences the ThESC migration and invasion capacities

**Figure 4:**
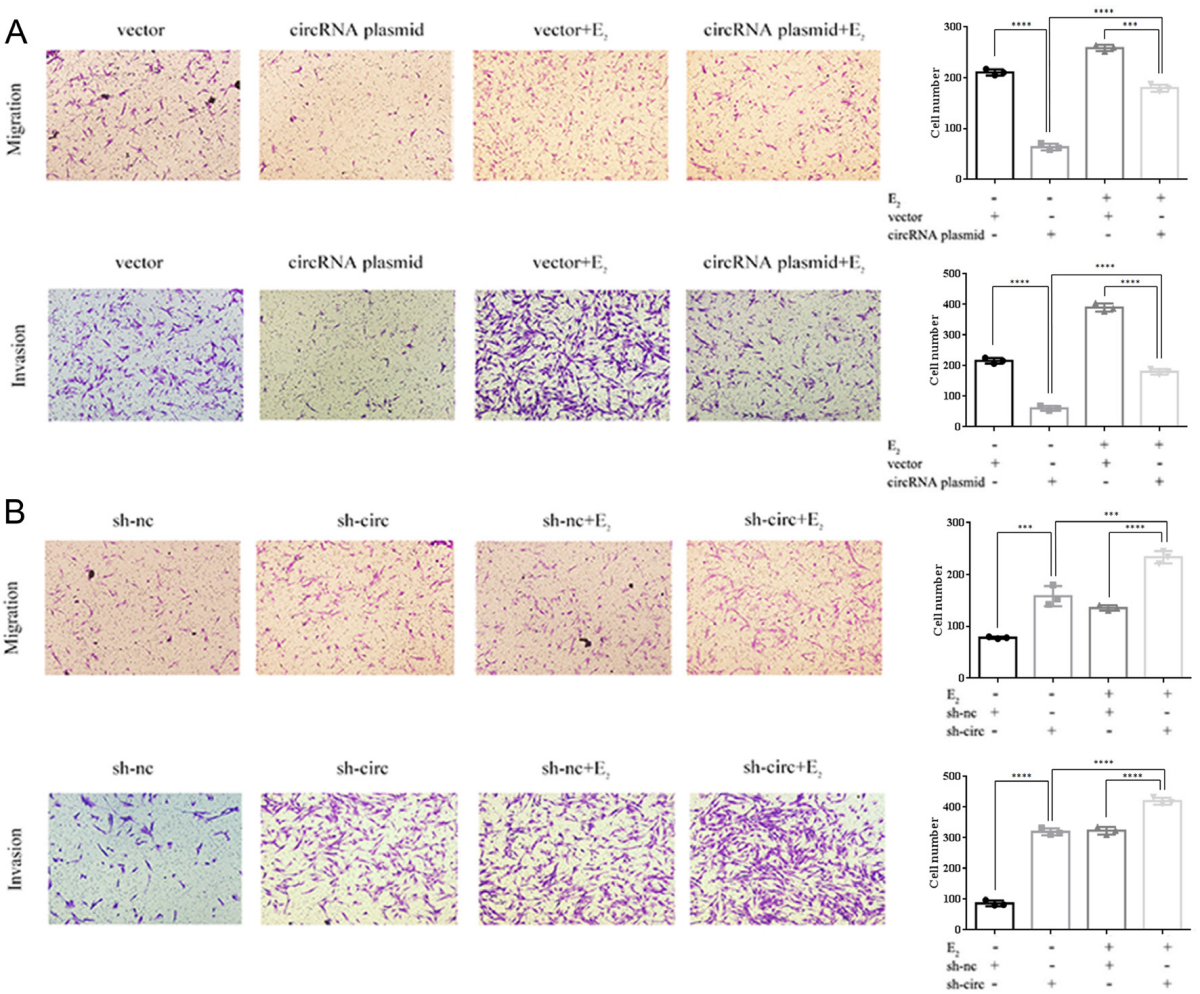
Hsa_circ_0001649 influences the migration and invasion capacities of ThESCs. (A) Transwell assays showed the migration and invasion capacities of ThESCs transfected with vector, circRNA plasmid, vector + E_2_, and circRNA plasmid + E_2_. (B) Transwell assays showed the migration and invasion capacities of ThESCs transfected with sh-nc, sh-circ, sh-nc + E_2_, and sh-circ + E_2_. All images were taken at 200× magnification. The data are presented as the mean ± s.d. of three independent experiments. ***P < 0.0001 and ****P < 0.0001 by one-way ANOVA.

Considering the downregulation of hsa_circ_0001649 in EMs and its relationship with MMP9, determining its functional role was necessary. Therefore, transwell assays were used to detect the invasion and migration abilities of ThESCs. As shown in Fig. 4, E_2_ promoted the migration and invasion abilities of ThESCs, while overexpression of hsa_circ_0001649 reduced the migration and invasion of ThESCs and even reversed the promotional ability of E_2_ (Fig. 4A). Correspondingly, the knockdown of hsa_circ_0001649 had the opposite effect (Fig. 4B). These results shed light on the metastasis-promoting role of hsa_circ_0001649 downregulation in ThESCs.

## Discussion

In the present study, we evaluated hsa_circ_0001649 expression in women with EMs and found that, compared with normal endometrial tissue, ectopic and eutopic endometrial tissues from EMs patients had lower hsa_circ_0001649 levels. Moreover, hsa_circ_0001649 was involved in the E_2_-mediated upregulation of MMP9 in ESCs and ThESCs, suggesting that hsa_circ_0001649 functions as a migration–invasion suppressor in EMs, which is consistent with its function in colorectal cancer, gastric cancer and hepatocellular carcinoma (Qin* et al.* 2016, Li* et al.* 2017*a*, Ji* et al.* 2018).

CircRNAs represent newly discovered star RNAs. Currently, research investigating circRNAs is insufficient and many problems remain to be solved. Two outstanding problems include the identification of factors that regulate circRNAs and the mechanism by which circRNAs exert physiological and pathological effects. Therefore, our finding of differential hsa_circ_0001649 expression in EMs represents an initial step toward identifying the factors underlying decreased hsa_circ_0001649 expression. Studies have shown that, in clear cell renal cell carcinoma, breast cancer and granulosa cells, the expression levels of circRNAs are associated with E_2_ or Ers (Huang* et al.* 2018, Klinge 2018, Qiao* et al.* 2018). Because E_2_ plays key roles in Ems (Huhtinen* et al.* 2012), we suspected and further confirmed experimentally that E_2_ can inhibit circRNA expression in ESCs through ERs. However, further studies are needed to determine which ER plays a role in the regulatory mechanism. Although most studies have reported changes only in downstream circRNA expression during E_2_ action or ER knockout (Nair* et al.* 2016, Li* et al.* 2017*b*, Song* et al.* 2019, Yuan* et al.* 2019), one study showed that, in bladder cancer, ERα can regulate the expression of circ_0023642 by regulating the expression of its host gene UVRAG at the transcriptional level (Wu* et al.* 2019). We used JASPAR (http://jaspar.genereg.net/), a web-based approach, to predict potential E_2_ response elements (EREs) in the promoter region of the hsa_circ_0001649 host gene SHPRH and identified several potential ERβ binding sites. ChIP and dual-luciferase assays can be used to verify whether ERβ has a direct regulatory effect on SHPRH. Moreover, the RNA-binding protein Quaking (QKI) regulates circRNA formation during EMT by binding sites flanking circRNA-forming exons and inducing exon circularization (Conn* et al.* 2015). Notably, hsa_circ_0001649 is strongly regulated following EMT (Conn* et al.* 2015), which indicates that QKI directly binds to the host gene SHPRH to promote hsa_circ_0001649 formation in EMs. Moreover, the information in the JASPAR database shows that QKI does not have an ERα binding site, but it does have an ERβ binding site in the upstream promoter region, suggesting that it may be regulated by ERβ. Studies have reported that higher ERβ levels and enhanced ERβ activity are detected in endometriotic tissues (Han* et al.* 2015). Therefore, whether ERβ can regulate QKI or directly binds to SHPRH to reduce hsa_circ_0001649 expression in EMs is worthy of further investigation.

We next studied the function of hsa_circ_0001649 and revealed that it increases MMP9 expression and affects invasion and metastasis in ESCs, but we did not further study the specific mechanism by which hsa_circ_0001649 impacts MMP9. Furthermore, the functions of circRNAs remain largely unknown. It has been proposed that circRNAs may act as competing endogenous RNAs (ceRNA) to miRNAs (miRNA sponges). The two most widely known examples are ciRS-7/CDR1as, which functions as a miR-7 sponge, and sex-determining region Y (Sry) 9, which functions as a miR-138 sponge (Huhtinen* et al.* 2012, Klinge 2018). We found that hsa_circ_0001649 has the capacity to harbor miRNA binding sites and possesses potential binding sites for hsa-miR-1231, hsa-miR-223 and 20 other miRNAs (https://circinteractome.nia.nih.gov/miRNA_Target_Sites/mirna_target_sites.html). Based on CircInteractome (a web tool that can explore potential miRNAs interacting with circRNAs), we found that hsa_circ_0001649 has the capacity to harbor miRNA binding sites and possesses potential binding sites for hsa-miR-1231, hsa-miR-223 and 19 other miRNAs. Further prediction based on TargetScan (miRNA target prediction software that uses the same algorithm as CircInteractome) revealed that hsa_circ_0001649 has four binding sites for hsa-miR-203a, two binding sites for hsa-miR-127, hsa-miR-331, hsa-miR-486, hsa-miR-488, hsa-miR-545, hsa-miR-649, and hsa-miR-942 and one binding site for 13 others miRNAs (Supplementary Table 2) (Dudekula* et al.* 2016). Therefore, hsa_circ_0001649 might play a role in EMs through interactions with miRNAs. However, other studies have suggested that few circRNAs contain a substantial number of miRNA binding sites for a single miRNA, and it remains to be clarified whether circRNA can function as a miRNA sponge (Ebbesen* et al.* 2016). A search for reiterated miRNA binding sites in circRNAs regulated by transforming growth factor β treatment inhuman mammary epithelial cells did not reveal any candidates (Conn* et al.* 2015). Similarly, a computational analysis of available circRNA sequences identified only two with more miRNA sites than expected by chance (Guo* et al.* 2014). Thus, RNA pull-down and other experiments are needed to further elucidate the mechanism of hsa_circ_0001649 and determine whether it functions as a miRNA sponge in EMs.

**Figure 5:**
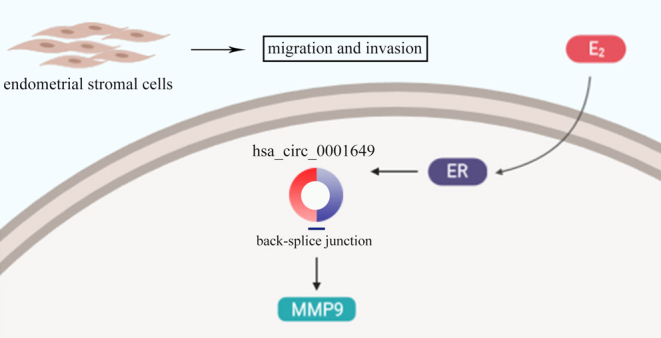
A schematic diagram depicting how estrogen affects migration and invasion by regulating hsa_circ_0001649 expression in human primary endometrial stromal cells (ESCs). The image was created with BioRender.com.

In summary, our results show that E_2_ decreased hsa_circ_0001649 expression through ERs, which further upregulated the expression of MMP9 and enhanced ESC migration and invasion (Fig. 5). These findings have improved our understanding of the molecular mechanisms underlying E_2_-mediated cell invasion in EMs.

## Supplementary Material

Figure S1. The relative positions of exons, the BSJ region and primers compared with hsa_circ_0001649.

Figure S2. Hsa_circ_0001649-knockdown plasmid construction. (A) Schematic of the shRNA-mediated circRNA interference pathway. (B) The control shRNA (the black dotted line) and the shRNA for hsa_circ_0001649 (the yellow line).

Figure S3. Schematic of circRNA overexpression by the hsa_circ_0001649 plasmid.

Supplementary Table 1. Primer sequences.

Supplementary Table 2. Potential miRNA-circRNA binding sites predicted by CircInteractome and TargetScan.

## Declaration of interest

The authors declare that there is no conflict of interest that could be perceived as prejudicing the impartiality of the research reported.

## Funding

This project was supported by the National Natural Science Foundation of Chinahttp://dx.doi.org/10.13039/501100001809 (Grant No. 81471439 Y L) and by Hubei Provincial Natural Science Foundation of China (2019CFB148).

## Author contribution statement

Q Li, Y Liu and L Zhang conceived the study and wrote the paper. N Li, H Liu, Y Du and H He performed the experiments and analyzed the data.
