# Supplementary figures and images for: Estrogen-decreased hsa_circ_0001649 promotes stromal cell invasion in endometriosis
Source: Reproduction. 2020 Jul 6;160(4):511–9. doi: 10.1530/REP-19-0540 (PMC7497355; doi:10.1530/REP-19-0540)

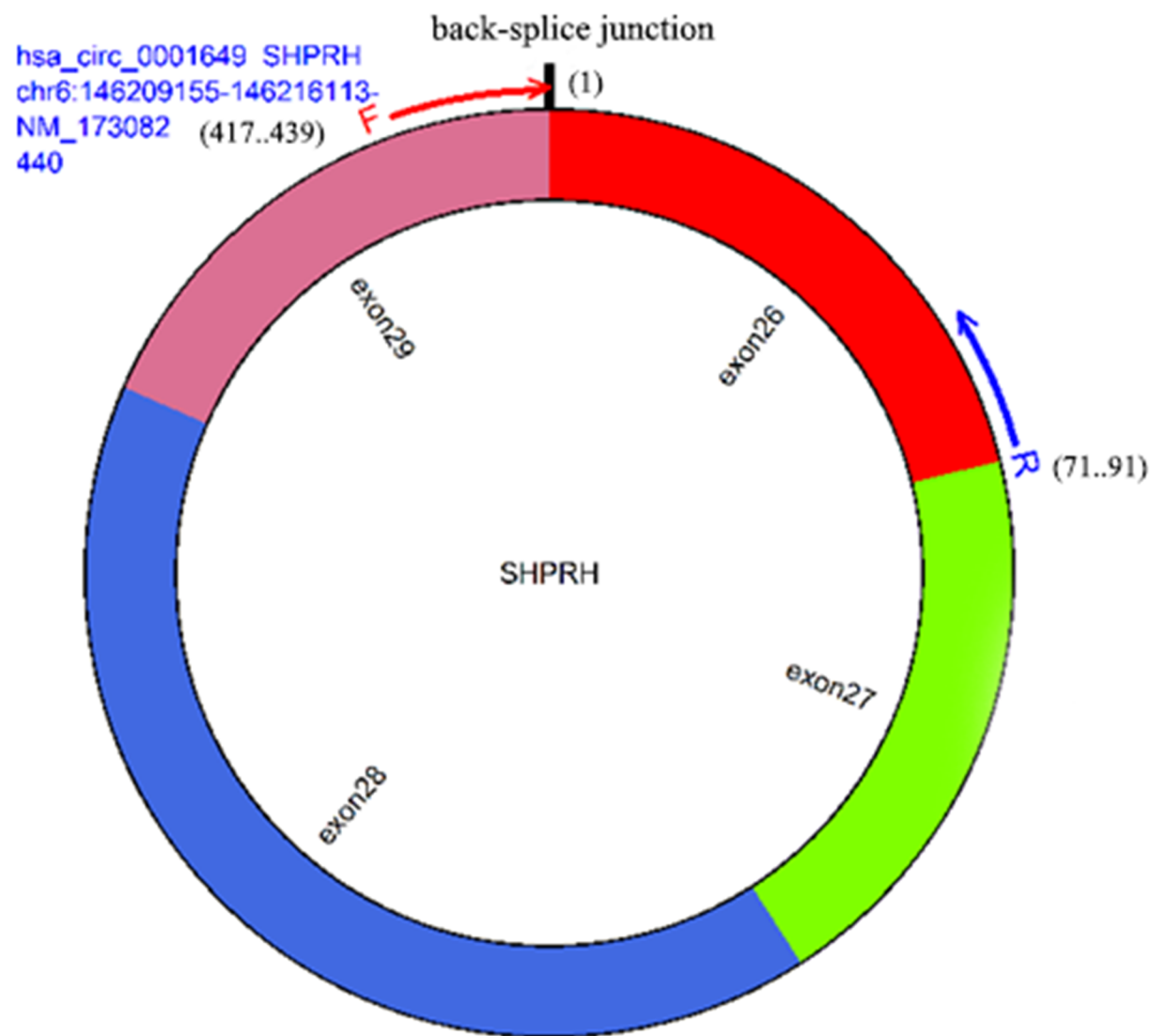

Supplement: Figure S1. The relative positions of exons, the BSJ region and primers compared with hsa_circ_0001649. [file supplementary_figure_1.pdf]

A

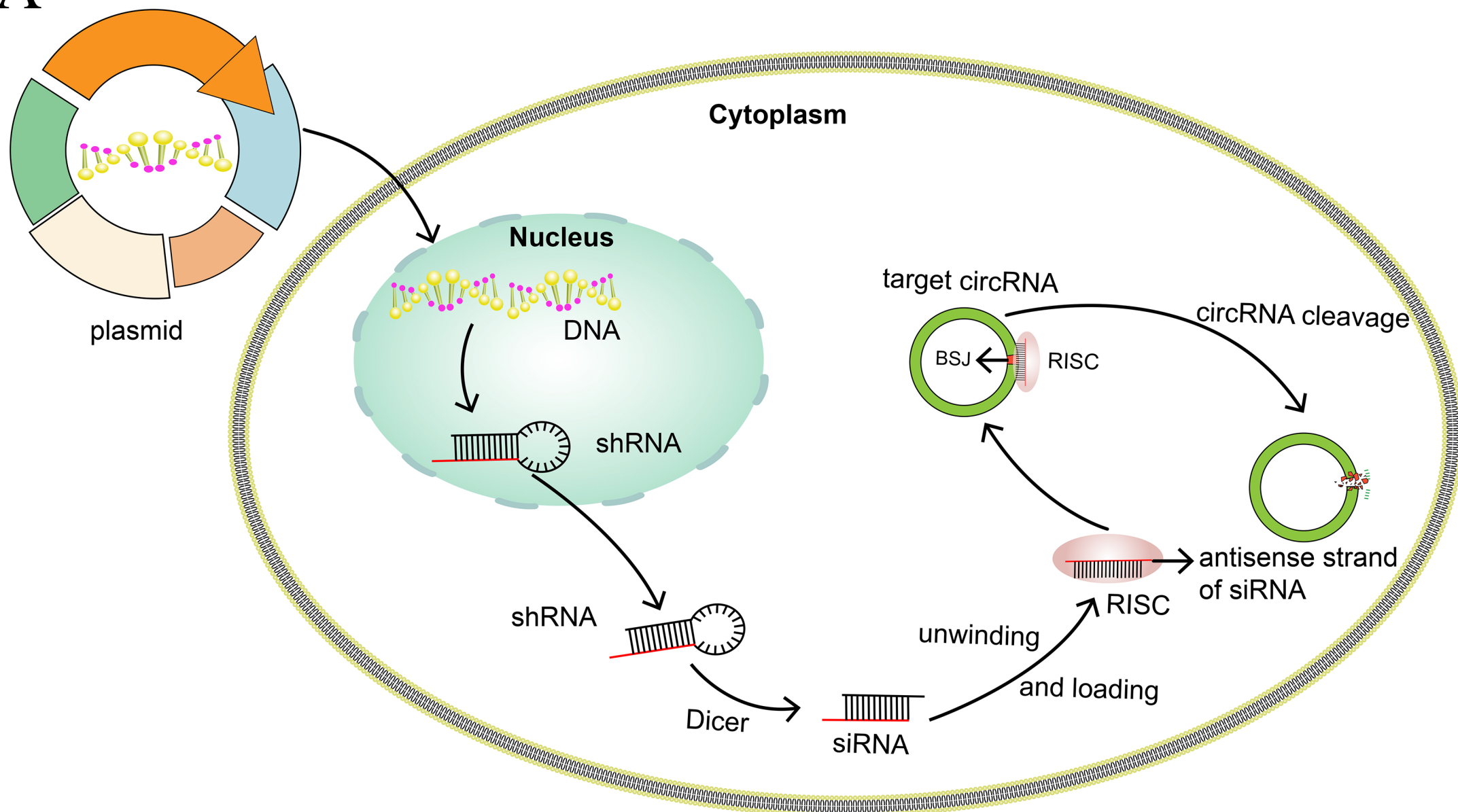

B

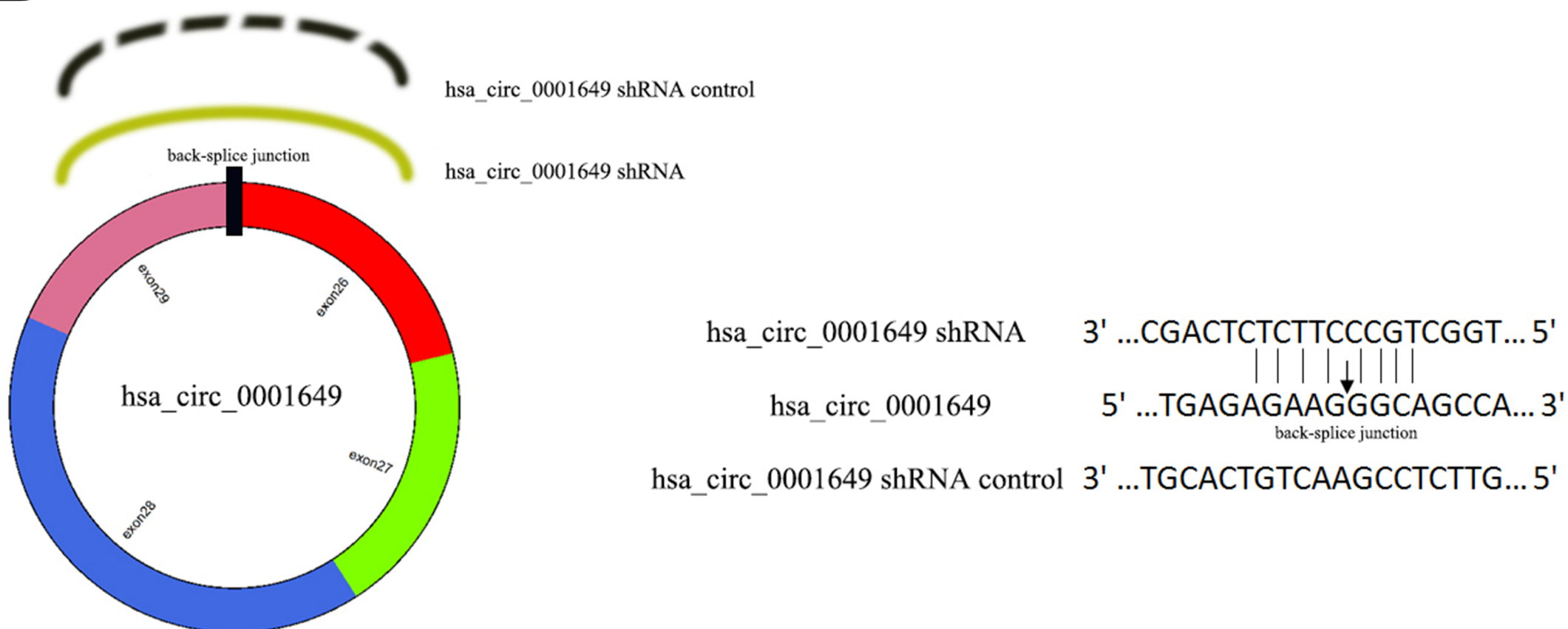

Supplement: Figure S2. Hsa_circ_0001649-knockdown plasmid construction. (A) Schematic of the shRNA-mediated circRNA interference pathway. (B) The control shRNA (the black dotted line) and the shRNA for hsa_circ_0001649 (the yellow line). [file supplementary_figure_2.pdf]
