# Supplementary material for: Estrogen-decreased hsa_circ_0001649 promotes stromal cell invasion in endometriosis
Source: Reproduction. 2020 Jul 6;160(4):511–9. doi: 10.1530/REP-19-0540 (PMC7497355; doi:10.1530/REP-19-0540)
Supplement: Figure S3. Schematic of circRNA overexpression by the hsa_circ_0001649 plasmid. [file supplementary_figure_3.pdf]

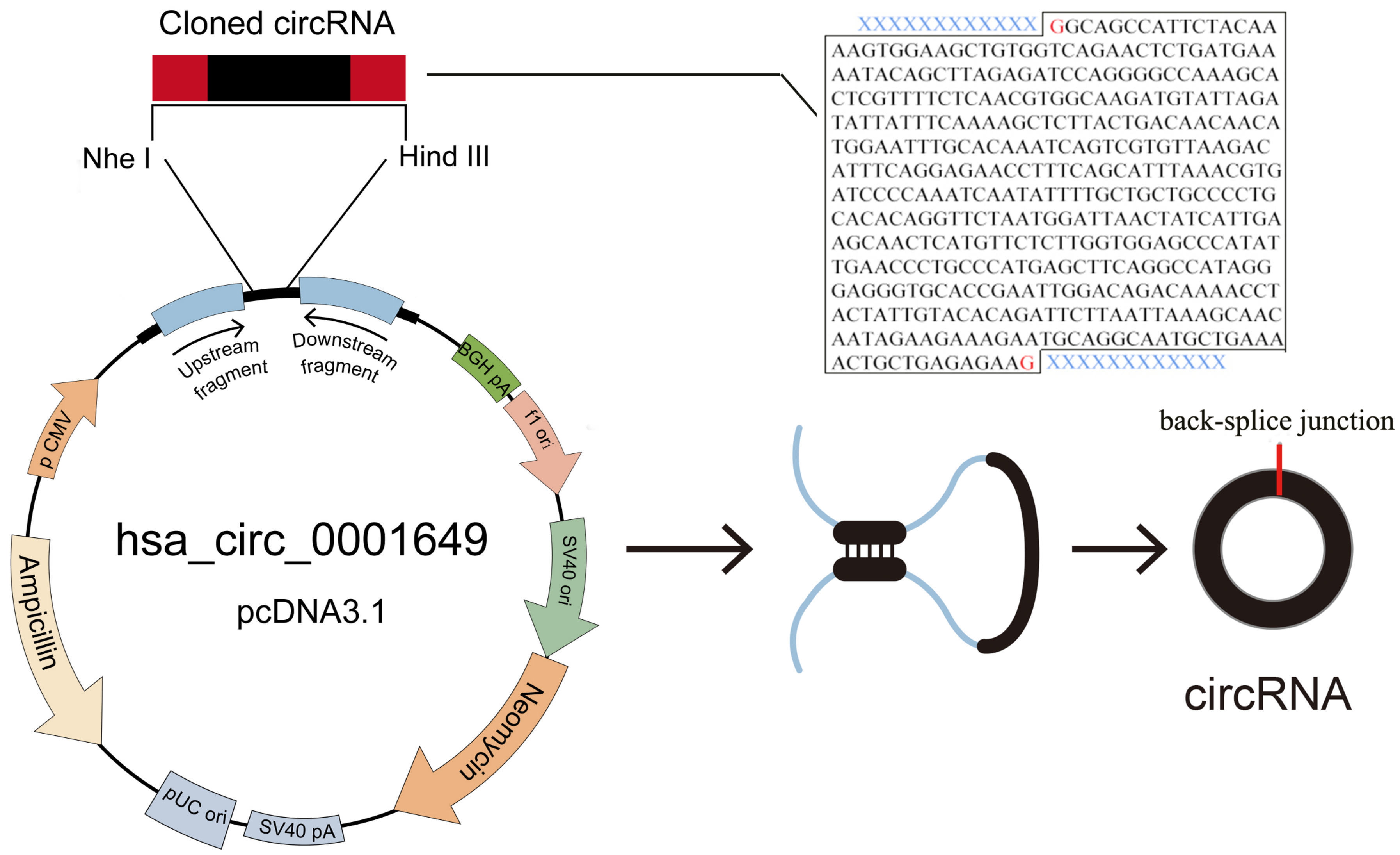
Cloned circRNA
Nhe I
Hind III
XXXXXXXXXXXX GGCAGCCATTCTACAA
AAGTGGAAGCTGTGGTCAGAACTCTGATGAA
AATACAGCTTAGAGATCCAGGGGCCAAAGCA
CTCGTTTTCTCAACGTGGCAAGATGTATTAGA
TATTATTTCAAAAGCTCTTACTGACAACAACA
TGGAATTTGCACAAATCAGTCGTGTTAAGAC
ATTTCAGGAGAACCTTTCAGCATTTAAACGTG
ATCCCCAAATCAATATTTTGCTGCTGCCCCTG
CACACAGGTTCTAATGGATTAACTATCATTGA
AGCAACTCATGTTCTCTTGGTGGAGCCCATAT
TGAACCCTGCCCATGAGCTTCAGGCCATAGG
GAGGGTGCACCGAATTGGACAGACAAAACCT
ACTATTGTACACAGATTCTTAATTAAAGCAAC
AATAGAAGAAAGAATGCAGGCAATGCTGAAA
ACTGCTGAGAGAAG XXXXXXXXXXXX
Upstream fragment
Downstream fragment
BGH pA
f1 ori
p CMV
hsa_circ_0001649
pcDNA3.1
SV40 ori
Ampicillin
Neomycin
pUC ori
SV40 pA
back-splice junction
circRNA
