# Supplementary material for: Estrogen-decreased hsa_circ_0001649 promotes stromal cell invasion in endometriosis
Source: Reproduction. 2020 Jul 6;160(4):511–9. doi: 10.1530/REP-19-0540 (PMC7497355; doi:10.1530/REP-19-0540)
Supplement: Supplementary Table 1. Primer sequences. [file supplementary_table_1.pdf]

Supplementary material

Supplementary Table 1. Primer sequences.

**Amplicon**	**Primer FW (5′–3′)**	**Primer RV (5′–3′)**
hsa_circ_0001649	AATGCTGAAAACTGCTGAGAGAA	TTGAGAAAACGAGTGCTTTGG
SHPRH	ATGAGCAGCCGACGGAAACG	ATCTGAACCTGGGCAGGGCT
MMP9	GCCTTCGCACTGTGGAGC	GGATACCCGTCTCCGTGCTC
GAPDH	TCAGGCGTCTGTAGAGGCTT	ATGCACATCCTTCGATAAGACTG
