# Supplementary material for: Estrogen-decreased hsa_circ_0001649 promotes stromal cell invasion in endometriosis
Source: Reproduction. 2020 Jul 6;160(4):511–9. doi: 10.1530/REP-19-0540 (PMC7497355; doi:10.1530/REP-19-0540)
Supplement: Supplementary Table 2. Potential miRNA-circRNA binding sites predicted by CircInteractome and TargetScan. [file supplementary_table_2.pdf]

Supplementary material

Supplementary Table 2. Potential miRNA-circRNA binding sites predicted by CircInteractome and TargetScan.

CircBase ID	miRNA ID	Binding site number
hsa_circ_0001649	hsa-miR-1231	1
hsa_circ_0001649	hsa-miR-1250	1
hsa_circ_0001649	hsa-miR-127-5p	2
hsa_circ_0001649	hsa-miR-140-3p	1
hsa_circ_0001649	hsa-miR-203	4
hsa_circ_0001649	hsa-miR-223	1
hsa_circ_0001649	hsa-miR-520f	1
hsa_circ_0001649	hsa-miR-331-3p	2
hsa_circ_0001649	hsa-miR-338-5p	1
hsa_circ_0001649	hsa-miR-486-3p	2
hsa_circ_0001649	hsa-miR-488	2
hsa_circ_0001649	hsa-miR-545	2
hsa_circ_0001649	hsa-miR-548p	1
hsa_circ_0001649	hsa-miR-571	1
hsa_circ_0001649	hsa-miR-580	1
hsa_circ_0001649	hsa-miR-604	1
hsa_circ_0001649	hsa-miR-647	1
hsa_circ_0001649	hsa-miR-649	2
hsa_circ_0001649	hsa-miR-885-5p	1
hsa_circ_0001649	hsa-miR-889	1
hsa_circ_0001649	hsa-miR-942	2
